# How Lipids Contribute to Autophagosome Biogenesis, a Critical Process in Plant Responses to Stresses

**DOI:** 10.3390/cells10061272

**Published:** 2021-05-21

**Authors:** Rodrigo Enrique Gomez, Josselin Lupette, Clément Chambaud, Julie Castets, Amélie Ducloy, Jean-Luc Cacas, Céline Masclaux-Daubresse, Amélie Bernard

**Affiliations:** 1 Laboratoire de Biogenèse Membranaire, UMR 5200, CNRS, Université de Bordeaux, F-33140 Villenave d’Ornon, France; rodrigo-enrique.gomez@u-bordeaux.fr (R.E.G.); josselin.lupette@u-bordeaux.fr (J.L.); clement.chambaud@u-bordeaux.fr (C.C.); julie.castets@u-bordeaux.fr (J.C.); 2 Institut Jean-Pierre Bourgin, UMR 1318 AgroParisTech-INRAE, Université Paris-Saclay, 78000 Versailles, France; amelie.ducloy@inrae.fr (A.D.); jean-luc.cacas@agroparistech.fr (J.-L.C.); celine.masclaux-daubresse@inrae.fr (C.M.-D.)

**Keywords:** autophagy, environmental stresses, lipids, ATG proteins, autophagosomes, ER-stress

## Abstract

Throughout their life cycle, plants face a tremendous number of environmental and developmental stresses. To respond to these different constraints, they have developed a set of refined intracellular systems including autophagy. This pathway, highly conserved among eukaryotes, is induced by a wide range of biotic and abiotic stresses upon which it mediates the degradation and recycling of cytoplasmic material. Central to autophagy is the formation of highly specialized double membrane vesicles called autophagosomes which select, engulf, and traffic cargo to the lytic vacuole for degradation. The biogenesis of these structures requires a series of membrane remodeling events during which both the quantity and quality of lipids are critical to sustain autophagy activity. This review highlights our knowledge, and raises current questions, regarding the mechanism of autophagy, and its induction and regulation upon environmental stresses with a particular focus on the fundamental contribution of lipids. How autophagy regulates metabolism and the recycling of resources, including lipids, to promote plant acclimation and resistance to stresses is further discussed.

## 1. Introduction

Plants are sessile organisms subject to a plethora of stresses during their life cycle. These stresses include abiotic stresses depending on climatic conditions, such as drought and osmotic stresses, nutritional starvation in macro and micronutrients, temperature, and intensity and quality of light, but also biotic stresses, including attacks by pathogenic and herbivory organisms and viruses. Additionally, phenology plays a critical role in the plant life cycle. These periodic developmental events, including seed formation, anthers dehiscence, pollen grain development, tuberization, flowering, leafing, and senescence, are dependent on environmental conditions and in fine on current global changes. To adapt to these wide range of environmental and developmental constraints, plants have developed sophisticated defense and quality control processes, including macroautophagy (self-eating), an intracellular mechanism mediating the degradation and recycling of intracellular compounds in eukaryotic organisms (mammals, fungi, and plants) [1]. 

During autophagy, portions of the cytoplasm are engulfed by a growing membrane structure which ultimately fuses to form a double membrane vesicle called autophagosome (AP). Upon completion, the AP traffics to the lytic vacuole and fuses with the tonoplast to deliver its content in the lumen where it will be rapidly degraded by vacuolar hydrolases [2]. Autophagy can randomly or selectively target specific components of the cell including proteins, protein aggregates, and damaged or unwanted organelles, and thus participates in the control of the cell quality and homeostasis. In recent years, increasing work has unraveled the critical contribution of autophagy in plants’ response to environmental changes. Kept at a basal level in optimal growing conditions, autophagy is induced by multiple stresses including nutrient starvation, drought, or heat stress during which the macromolecules resulting from autophagy degradation can be reused by the cell to support a basal metabolism and promote plant acclimation and resistance to environmental constraints [3]. Autophagy activity is also modulated throughout plant development with an increase during plant aging, as a critical process for nitrogen remobilization from senescing leaves to sink tissues [4,5,6]. 

**Figure 1:**
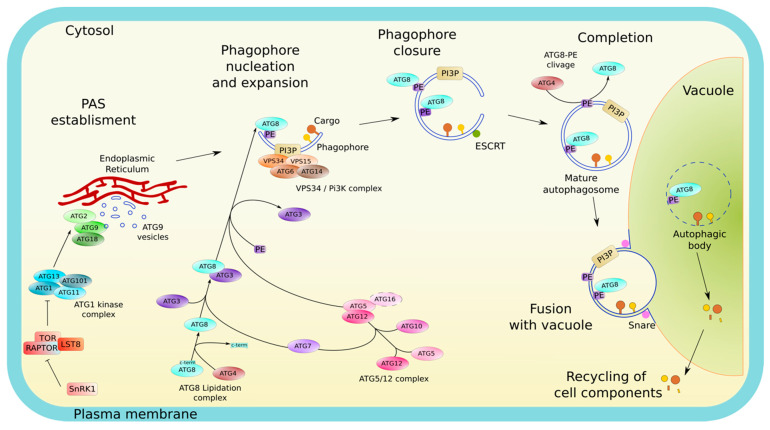
Overview of the autophagy pathway. In nutrient-rich conditions the master kinase TOR represses autophagy. Upon nutrient starvation, TOR activity is inhibited and autophagy is induced. Autophagosome (AP) biogenesis starts with the relocalization of the ATG1 initiation complex on specific subdomains of the ER used as platforms for the establishment of the pre-autophagomal structure (PAS), the lipo-proteic core from which the phagophore emerges. The ATG1 complex recruits further ATG proteins including the ATG9 vesicles and the autophagy-specific PI3K complex, leading to the nucleation of the double membrane phagophore. At the phagophore, an extensive machinery (consisting of the ATG5-ATG12/ATG16 complex, ATG10, ATG4, ATG3, and ATG7) mediates the covalent conjugation of ATG8 to a phosphatidylethanolamine (PE). The incorporation of ATG8-PE notably contributes to the expansion of the phagophore and the selection of autophagic cargo. Once the structure has reached its final size, the membranes at the rim of the phagophore eventually fuse to form a sealed AP in a process implicating ESCRT complexes. Upon AP completion, ATG proteins dissociate from its outer membrane, which, for ATG8, requires the activity of the cysteine protease ATG4. Then, the outer membrane of the mature AP fuses with the vacuolar membrane resulting in the delivery and subsequent degradation by vacuolar hydrolases of the AP inner membrane (called « autophagic body» (AB) when present inside the vacuole) along with the autophagic cargo. Finally, the resulting macromolecules are recycled back into the cytosol.

Central to autophagy activity and its regulation is the AP, the specialized autophagy vesicle, which targets the cargo to be degraded and delivers it to the lytic vacuole. AP formation results from a multistep process governed by the core-component of the autophagy machinery, the AuTophaGy-related proteins (ATG in plants and mammals; Atg in yeast) and relies on multiple membrane remodeling events (Figure 1). (1) Upon autophagy induction ATG proteins are sequentially recruited at the pre-autophagosomal structure (PAS) on specific subdomains of the endoplasmic reticulum (ER); (2) at the PAS, the concerted action of ATG proteins promotes the nucleation of an initial membrane structure, the phagophore, an open cup-like double membrane, with highly curved edges; (3) the phagophore then expands by addition of proteins and lipids/membrane, surrounding the cargo; (4) membranes at the rim of the phagophore finally fuse thus completing the formation of the AP; (5) the outer membrane of the AP fuses with the vacuole which releases the inner membrane and the AP content in the form of autophagic bodies (AB) in the vacuolar lumen; and (6) the cargo is degraded by hydrolases (lipases, proteases); breakdown products are then recycled into the cytosol in building blocks (free fatty acids, amino acids, sugars) where they re-enter metabolic pathways (Figure 1) [7]. 

The biogenesis of AP is thus a highly dynamic membrane-based process which induction and magnitude need to be very finely regulated in time and space to support cell quality and adaptation to stresses [8]. Among the molecular players of autophagy, the contribution of ATG proteins has been well studied. Recent advances in the field now support that membrane lipids are also fundamental components of the autophagy machinery. In fact, both quality and quantity of lipid are thought to be essential during stress-induced autophagy: (1) the nature of lipids composing AP membranes are key components of their structure and function with a potential contribution at each step of AP formation; and (2) availability of lipids as building blocks likely controls size and number of AP thus dictating rates of autophagy. 

In this review, we report our current knowledge on the implication of autophagy in plant response to stresses. How autophagy activity, induction, and magnitude is instructed and regulated is presented with a focus on the fundamental contribution of membrane lipids in this process. Finally, we discuss how, by rewiring of central cell activities upon stresses including lipid metabolism, autophagic degradation mediates plant acclimation to environmental changes.

## 2. Autophagy, a Series of Intense Membrane Remodeling Events Controlling Plant Responses to Environmental Stresses

### 2.1. Morphology and Membrane Dynamics in Autophagy

APs are central structures which play essential roles in the autophagy pathway by targeting and engulfing cargo prior to its delivery to the vacuole for degradation. APs are unique vesicles in the endomembrane system; first because they are made of a double lipid bilayer, second because they do not bud from a pre-existing membrane. Instead, they form *de novo* in a multistep process that requires numerous membrane remodeling events. 

Upon induction, autophagy is initiated by the recruitment of the core autophagy machinery composed of ATG proteins [9] from the cytoplasm, where they reside at steady state, to the PAS, an initial lipo-proteic condensate which defines the site of AP formation [10] (Figure 1). Unlike yeast cells, which display a unique PAS close to the vacuole [11], mammals show multiple PAS that seem to emerge from phosphatidylinositol-3-phosphate (PI3P) enriched subdomains of the ER [12]. Likewise, plants exhibit multiple PAS that form in close apposition to specific subdomains of the ER [13,14]. In both plants and mammals, membrane contact sites between the ER and the plasma membrane (PM) have also been implicated in early stages of AP biogenesis [15,16]. At the PAS, the sequential and coordinated actions of ATG proteins orchestrate the assembly and expansion of the phagophore (Figure 1).

The exact mechanism of phagophore nucleation is not well understood, although multiple hypotheses exist concerning the source of this initial membrane. The ATG1 kinase complex (ULK1 in mammals) is part of the autophagy initiation complex and regulates the activity and the recruitment of downstream ATG proteins required for AP biogenesis. ATG9, the only transmembrane protein among ATGs, resides in vesicles called “ATG9 vesicles” that, upon autophagy induction, are recruited by the ATG1 complex to the PAS [17,18] in yeast and mammals. ATG9 vesicles and other vesicles such as endosomes or the ER exit sites (ERES)-derived COPII vesicles coalesce at the PAS and are thought to serve as membrane seeds for the nucleation of the initial phagophore membrane [19,20,21,22,23]. However, in plants, ATG9 seems to have a distinct function than that of its yeast and mammalian counterparts. In Arabidopsis, ATG9 is not strictly required for the formation of autophagic structures suggesting that ATG9 may have alternative or additional functions in plants compared to other organisms. Nevertheless, the *atg9* knock-out mutant display large abnormally shaped autophagy structures and shows reduced rates of autophagy flux indicating that the ATG9 protein has a major function in proper AP formation [24]. 

The ATG1 complex is also implicated in the activation of the autophagy specific PI3 kinase complex (PI3K) [25]. The PI3K complex mediates the production of PI3P at the PAS and further recruit PI3P-effectors, such as ATG18 [26], and the ATG12-ATG5-ATG16 complex [27]. The local enrichment of PI3P at the PAS is a hallmark of autophagy as it occurs in mammals, yeast, and plants. In plants, both the PI3K complex [28] and the ATG1 complex [29] have been identified and implicated in the autophagy pathway. Moreover, components of both complexes were also found to be degraded via autophagy indicating that a tight regulation loop could limit the extent of autophagy activity in plant cells [28,29]. Interestingly, mutants of the initiation complex were still capable of producing the ATG8-phosphatidylethanolamine (PE) conjugate, although they did not form APs [29]. This could mean that in plants, the initiation complex acts in stages succeeding ATG8 lipidation, i.e., in latter stages of AP formation. 

Upon its assembly, the phagophore expands by addition of lipids and membranes while keeping its unique structure and ensuring the engulfment of cargo (Figure 1). Morphologically, the phagophore is observed as a cup with a highly curved rim. Curvature at the rim is thought to be a critical structure involved in the expansion of the phagophore which notably relies on the association of peripheral membrane proteins (including ATG proteins). Among those, several proteins sense membrane curvature as cues for their recruitment to this early autophagy structure [30]. In fact, ATG3 and ATG5 were shown to localize precisely at the rim of the phagophore to support the localized conjugation of ATG8 to PE [31], a prerequisite for its incorporation into autophagic structures and a key step in phagophore elongation, cargo selection and AP formation [31,32]. 

Once the phagophore has reached its final size, the membranes at the rim of the phagophore fuse together, sealing the structure that becomes a double membrane vesicle, the AP. The sealing of the phagophore structure is directed by the ENDOSOMAL SYSTEM COMPLEX REQUIRED FOR TRANSPORT (ESCRT) in mammals [33,34] and in plants [35]. Upon completion, many ATG proteins dissociate from the AP and are recycled back to the cytosol [36]. From then, mature APs dissociate from the ER and traffic towards the vacuole for fusion. The outer membrane of APs fuses with that of the vacuole which results in the delivery of the internal membrane along with the cargo inside of the vacuole for degradation. To allow proper fusion, a tether between APs and the vacuole/lysosome membranes is required. Multiple SNARE (SOLUBLE N-ETHYLMALEIMIDE-SENSITIVE FACTOR ATTACHMENT PROTEIN RECEPTORS) complexes have been implicated in this process in yeast and mammals (reviewed in [37,38]). In plants, the molecular machinery implicated in this process is not well characterized, but some actors have been identified. The FYVE DOMAIN PROTEIN REQUIRED FOR ENDOSOMAL SORTING 1 (FREE1), which is a member of the ESCRT system is thought to participate in the fusion of APs to vacuoles [39]. Additionally, the VESICLE TRANSPORT V-SNARE 11–13 (VTI (11–13)) proteins, homologs of proteins of the mammalian SNARE complex, have been identified in Arabidopsis, and their mutants displayed phenotypes reminiscent of autophagy mutants, such as accelerated senescence suggesting a possible role of this complex in plant autophagy [40].

In conclusion, the AP biogenesis process requires intense membrane remodeling events to (1) nucleate a membrane de novo, (2) shape the membrane to a specific architecture, (3) expand this membrane by articulating multiple sources of lipid delivery while keeping structure and function, (4) regulate the formation and dissociation of membrane contact sites, (5) control membrane fusion with the rim of the phagophore and then with the tonoplast in a timely manner to ensure appropriate size of the AP and avoid premature fusion with the vacuole thus ensuring delivery of cargo for degradation. Further, by promoting the expansion/shaping of the phagophore, these events also control the size and the number of APs thereby modulating the rate and amplitude of autophagic activity. In the last 10 to 20 years, knowledge of the autophagy pathway, and specifically the analyses of mutants of the autophagy machinery (*atg* mutants), which, for the most part, show a complete block in autophagy activity, identified critical steps in the morphology of autophagy pathway and unraveled the critical contribution of autophagy in plant responses to environmental stresses that is presented below (also see Table S1).

### 2.2. Autophagy Is Critical for Plant Response to Abiotic and Biotic Stresses and Viruses

#### 2.2.1. Nutrient Starvation

To support their optimal development, plants need to draw essential nutrients from the soil, including macronutrients (N, P, S, Mg, Ca) and micronutrients (Fe, Zn, Cu, Ni, Mo, Mn, Cl, B). However, due to their inability to move, plants can have nutrient-deficient phases of their life cycle when the soil is poor [7]. In that context, the first studies focusing on the relationship between abiotic stresses and autophagy were carried out upon nutrient deficiencies showing that autophagy-impaired mutants are hypertensive to these conditions. In fact, the *atg9-1* mutant exhibits a chlorosis phenotype in the cotyledons and in the rosette leaves in comparison with the WT in nitrogen and carbon starvation [41]. Similar results were also obtained with the *atg7-1* mutant [42], *atg4a4b-1* [43], *atg5-1* [44], *AtATG6-AS* [45], *atg7-2* [46], *atg9-4* [47], *atg10-1* [48], *atg11-1* and *atg11-2* [46], *atg12a-1/atg12b-1* [49], *atg13a-1/atg13b-2* and *atg13a-2/atg13b-2* [29], and *atg18a* [50] mutants under nitrogen and carbon depletion. Recently, Shinozaki and collaborators, showed in particular that the *atg5* and *atg7* mutants presented difficulties to grow and an advanced chlorosis phenotype in zinc deficient condition [51].

#### 2.2.2. Drought and Osmotic Stress

In the last years, the durations of drought and their severity have been gradually increasing with global warming. Thus, understanding the responses of photosynthetic organisms to osmotic and drought stress has become a major issue for the scientific community and for the agriculture world. At the end of the 2000s, initial results highlighted the importance of autophagy in plants following osmotic stress. Simply put, plants with a reduced or absent autophagy activity mostly show an increased sensitivity to drought. In fact, the RNAi ATG18a mutant, the *atg5* and *atg7* mutants as well as the KO mutant for *NRB1* (*NEXT TO BRCA1 GENE 1*), a receptor for selective autophagy, all exhibit hypersensitivity to salt and drought treatments [52,53]. The *atg2*, *atg5*, and *atg7* mutants also show reduced germination in the presence of mannitol and the percentage of fresh weight, reduced sugars, and total soluble sugars decreased in these *atg* mutants after treatment with salt [54]. Similarly, the knock-down of ATG8-INTERACTING PROTEIN (ATI1) exhibited an increased sensitivity to salt suggesting its implication in the elimination of damaged proteins during salt stress [55]. In contrast, the *atg9* mutant and an ATG8 overexpressor line which shows higher autophagy flux, where both found to germinate more quickly than WT [54].

In contrast to autophagy mutants, plants with higher autophagy flux mostly show an increased resistance to water scarcity. For example, the overexpression of KIN10 (SNF1-RELATED PROTEIN KINASE CATALYTIC SUBUNIT ALPHA), which induces autophagy, promotes drought tolerance. Similarly, the overexpression or heterologous expression of various ATG8 homologues in Arabidopsis was shown to lead to a better plant response to salt and/or drought conditions. An Arabidopsis ATG8 overexpressor line which shows higher autophagy flux was shown to germinate more quickly than WT [54]. Heterologous expression of ATG3a and ATG3b from *Malus domestica* in Arabidopsis enhanced tolerance to salt and mannitol with a higher fresh weight and longer roots compared to WT [56]. Likewise, heterologous expression of ATG18a from apple (MdATG18a) in tomato plants (*Solanum lycopersicum*) or ATG8f from *Musa acuminata* (banana) in Arabidopsis improved drought stress tolerance [57]. Recently, Bao and colleagues identified a novel plant-specific autophagy regulator called COST1 (CONSTITUTIVELY STRESSED 1) which, in optimal environmental conditions, represses autophagy by directly interacting with ATG8 [58]. Upon water stress, COST1 is degraded, which induces autophagy. The *cost1* mutant, which shows higher autophagy, exhibits a better drought resistance phenotype and the authors showed that this is dependent on an active autophagy pathway. Overall, the authors propose that COST1 acts as a thermostat to mediate the balance between plant growth and plant stress tolerance [58].

#### 2.2.3. Heat Stress

In addition to nutritional deficiencies and osmotic/water stresses, plants are also subject to rather significant temperature variations depending on the season and even on the time of day with high differences between dawn, zenith sun, and dusk. Furthermore, with the escalation of global warming, the time of exposure to either low or high temperature is likely to increase during the 21st century with potential negative effects on crop productivity. For example, high temperatures promote the denaturation and misfolding of proteins inducing their degradation via quality control pathways such as autophagy. In fact, the *atg5-1*, *atg7-2,* and *nbr1-1* mutants are all hypersensitive to heat stress (37 °C or 45 °C) [53,59]. In comparison, the *atg2-1* mutant shows a only slight phenotype at 30 °C with a lower fresh weight and chlorophyll amount compared to WT [60]. Nevertheless, the *atg2-1* mutant and the *atg5-1*, *atg7-2,* and *atg10-1* mutants all show an alteration in pollen development and anthers dehiscence after treatment at 30 °C [61]. Recently, Thirumalaikumar and colleagues unraveled part of the molecular actors involved in the autophagy-dependent regulation of heat stress. The authors showed that NBR1 mediates the degradation of HSP90.1 (HEAT SHOCK PROTEIN 90.1) and ROF1 (ROTAMASE FKBP 1) via autophagy. In that manner, NBR1 is involved in the repression of the heat stress response by lowering the expression of *HSP* genes regulated by the transcription factor (TF) HSFA2 (HEAT SHOCK FACTOR A2) [62,63,64]. The same team also showed that autophagy was responsible for the accumulation of proteogenic dipeptides in response to heat stress [65]. Finally, to our knowledge, very little is currently known on the study of autophagy in cold stress conditions [66] which therefore constitutes an interesting line of research for a better understanding of the mechanisms linking abiotic stresses and autophagy.

#### 2.2.4. Light Modulation

During their life cycle, plants are also subjected to longer or shorter photoperiods depending on the seasons but also to variable light spectral qualities including visible light (400–700 nm) and ultraviolet-B (UV-B; 280–315 nm), the most energetic wavelength light reaching the surface of the Earth [66,67]. A strong and prolonged exposure to either visible light or UV-B can lead to large damage of plants macromolecules (DNA, proteins and lipids). Recently it was shown that the *atg2*, *atg5*, *atg7*, *atg10*, *atg12a,* and *atg18a* mutants were hypersensitive to a treatment with UV-B leading to a half reduction in the activity of PSII in comparison with the WT [66,67]. A high visible light treatment also induced a necrosis phenotype in the *atg2-1* mutant [60]. In addition, several autophagy mutants, including *atg7-1* [42], *atg5-1* [44], and *atg13a/atg13b* [29], exhibit accelerated senescence of their rosette leaves when cultivated in short days suggesting the importance of autophagy in the management of cell homeostasis and plant development upon different photoperiods.

#### 2.2.5. Biotic Interactions and Viruses

Throughout their development, plants have to face diverse types of harmful invaders, including bacteria, fungi, and viruses. Although autophagy has been involved in plant immunity, its function cannot be generalized because it can act either as a pro- and/or anti-pathogen process depending on the kind of pathogen, its lifestyle and its infection strategy [68]. While necrotrophs (such as *Botrytis cinerea* or *Alternaria brassicicola*) kill their host to obtain nutrients from dead tissue, biotroph pathogens (including particularly mildew like *Golovinomyces cichoracearum*) need a living host to proliferate inside [68]. These two strategies partially explain the duality of autophagic activity in the infected host, leading, in one case, to a beneficial impact and in the other to a detrimental effect.

Among necrotrophs, Wang and colleagues reported that the autophagy mutant *atg2-2* has a better resistance to powdery mildew *G. cichoracearum* than wild-type plants, but the mutant loses its resistance when key actors of salicylic acid (SA) pathway are mutated [69]. Similarly, the *atg2* mutant displays higher sensitivity to the necrotrophic bacteria *Dickeya dadantii*, but that sensitivity is abolished when the SA pathway is blocked [70]. Likewise, the *atg7* and *atg8a* mutants are more sensitive to infection with the non-pathogenic *Sclerotinia sclerotiorum* strain A2 than the control [71]. The majority of plant’s activities are regulated by signaling hormones (such as SA, jasmonic acid (JA), abscisic acid (ABA) or ethylene). Among those, SA plays critical roles in the defense responses against biotrophic pathogens [72] which can be either dependent or independent from its potential negative role of autophagy [69]. Studies of Arabidopsis *atg5*, *atg7,* and *atg18* mutants suggested that autophagy plays a positive role in plant resistance to fungal pathogens [73]. Blocking autophagy in KO mutants was shown to cause hypersensitive responses (HR) to necrotrophic fungi [74] and promoting the phosphorylation of ATG18a, which results in reduced autophagy activity, decreased plant resistance against *Botrytis cinerea*. Conversely, overexpressing a hyper-autophagy inducing mutant of ATG18a enhances resistance against this pathogen [74]. Note that certain kind of necrotrophic pathogens can hijack autophagy to their advantage. For example, the effector PexRD54 from the potato blight pathogen *Phytophthora infestans* binds the autophagy protein ATG8 to stimulate AP formation in order to eliminate defense-related compounds. Thus, pathogens can activate autophagy for their own benefit [75].

Among the biotrophic pathogens, the most studied example is *Pseudomonas syringae pv tomato* (*Pst*). Üstün and coworkers, identified both pro- and antibacterial functions of autophagy upon infection of *Arabidopsis thaliana* with *Pst*, a biotrophic pathogen which manipulates and modulates autophagy machinery via various effectors [76]. On the one hand, *Pst* can activate autophagy with the effector HrpZ1 to enhance autophagy through the modulation of ATG8 cleavage by ATG4. On the other hand, *Pst* can also negatively regulate autophagy with the effectors HopF3 and AvrPtoB by targeting the ATG8 and ATG1 module of the autophagy machinery, respectively [76].

Plants can also use autophagy as mean of defense to viruses. Autophagy deficient *Arabidopsis thaliana* mutants *atg5 and atg7* and the cargo receptor mutant *nbr1* exhibited stronger symptoms to *Cauliflower Mosaic virus* (CaMV) infection compared to WT [77] suggesting a protective role of autophagy against this virus. The authors have identified that P4, a major structural protein of viral capsid and assembled P4-viral-capsides are selectively targeted by NBR1 to mediate their degradation and restrict CaMV infection [77]. Similarly, to cope with RNA virus, plants use autophagy to target and degrade the RNA-dependent RNA polymerase. Li and colleagues have shown that infection by the *Turnip mosaic virus* (TuMV) activates autophagy in *N. benthamiana* [78]. In that study, the protein BECLIN1/ATG6 was found to interact specifically with Nib, the only RNA-dependent RNA polymerase of TuMV. The further interaction of BECLIN1/ATG6 with ATG8a triggers Nib degradation by autophagy thus inhibiting viral proliferation [78]. Similarly, in *N. benthamiana*, autophagy was found to mediate the degradation of the regulator of gene silencing calmodulin-like protein (rgs-CaM) together with the two viral effectors, HC-pro and 2b, involved in the suppression of the plant RNA silencing machinery which mediates defense against various viruses [79].

While plants can use autophagy for defense against viruses, the co-evolution of those pathogens and plants led to the establishment of counter-defense strategies by viruses, notably by suppressing autophagy. For example, the genome of the *Barley stripe mosaic virus* (BSMV), a RNA virus [80], encodes the γb protein which functions as a viral suppressor of RNA silencing and a modulator of host defences [81]. The BSMV γb protein was found to physically interact with ATG7 thus competing with the ATG7/ATG8 interaction, an essential component of AP formation. Thus, by disrupting the interaction between ATG7 and ATG8, γb inhibits autophagy and promotes viral infection.

#### 2.2.6. ROS and Autophagy

All of the stresses presented above can also result in the production of reactive oxygen species (ROS) that will act as a defense-signaling molecule for the plant and allow the establishment of a defense pathway involving the 26S proteasome and/or autophagy. Xiang and colleagues showed that autophagy is induced in wild-type *Arabidopsis thaliana* plants after transfer of seedlings to MS medium containing either hydrogen peroxide or methyl viologen (MV) [82]. In addition, the RNAi AtATG18a mutant exhibits hypersensitivity to oxidative stress [82,83]. Likewise, the *atg5*, *atg7,* and *nbr1* mutants also present hypersensitivity to oxidative stress after treatment with MV [53] whereas the overexpression of *ATG5* and *ATG7* leads to enhance resistance to MV [3]. Recently, both programmed cell death (PCD) and ROS level were reported to be regulated by autophagy during a compatible interaction between a plant and a virus [84]. Moreover, treatment of these plants with an autophagy inhibitor increases PCD, but not ROS level, systemically (not at infection site). This suggests that ROS act as signalling molecules and are produced in excess upon infection. In these conditions, autophagy is induced to eliminate the excess of ROS and/or to degrade damaged components (caused by ROS excess), thus protecting cells, where ROS are not needed to trigger PCD [84]. Recently, Chen and colleagues have also shown that autophagy is activated in *Arabidopsis thaliana* following hypoxic stress by submergence via the accumulation of ROS [85].

To conclude, converging elements based on the analyses of autophagy mutants support that autophagy mostly acts as a pro-survival mechanism upon a wide range (if not all) of abiotic stresses and during certain types of biotic or viral interactions. Accordingly, studies of plants overexpressing *ATG* genes support a beneficial impact of increasing autophagy activity which was associated with an enhanced resistance to drought [57], oxidative stress or necrotrophic pathogens [3]. Further, plants with higher constitutive autophagy exhibited delayed aging, and an enhanced plant growth and seed oil content suggesting better agronomical values (in both Arabidopsis [3] and rice [86]). Although this work opens the idea of manipulating autophagy activity as a way to increase crops tolerance to stress, at present, studies describing the effects of increasing or constitutively activating autophagy remains scarce. Thus, a direct correlation between autophagy activity and plant tolerance to a larger panel of environmental conditions should not be extrapolated. Notably, unchecked autophagy activity in mammals can also be detrimental for the cells and associated with muscular or neurodegenerative pathologies [87,88]. In plants, examples of excessive autophagy leading to detrimental phenotypes have not yet been reported to our knowledge. Reports of autophagy-mediated PCD exist in plants, yet this phenomenon was observed in response to a fungal infection and thus the PCD was proposed as a plant’s response mechanism to restrict the infection zone [71]. Finally, some pathogens can hijack autophagy to their advantages [89,90]. Therefore, a tight regulation of autophagy induction and amplitude in time and space is required to promote cell homeostasis during plant response to environmental stresses.

## 3. Membrane Lipids Are Fundamental Actors of Autophagy Activity, Induction, and Regulation upon Stress

**Figure 2:**
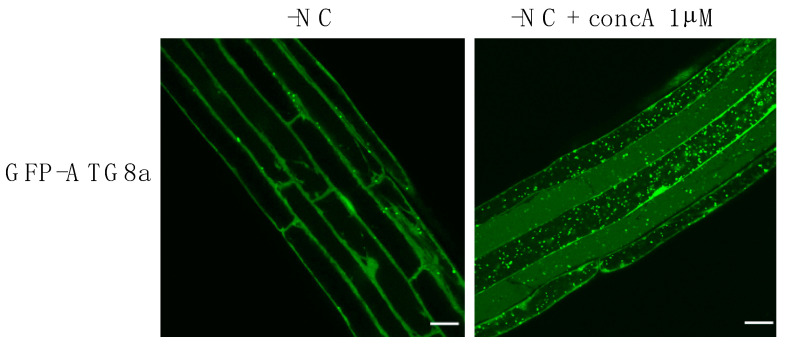
Blocking the degradation of autophagosomes (APs) reveal the tremendous amount of autophagy structures produced after 3 h of autophagy induction in Arabidopsis roots. Confocal microscopy images of GFP-ATG8a expressing plants. 7-day-old plantlets were transferred to liquid nutrient deprived medium (-NC) in control conditions (DMSO), or supplemented with Concanamycin A (-NC + concA 1 µM), for 3 h. Treatment with Concanamycin A inhibits vacuolar degradation thus resulting in the accumulation of autophagic bodies (ABs) inside the vacuole. Scale bar, 20 μm.

Maintaining the constants of the internal environment, also called homeostasis, is a complex process that requires multiple layers of regulation including the induction of autophagy and the control of its magnitude. The simplest and most classic way to follow the effect of environmental parameters on autophagy activity or autophagy flux is to observe the formation and degradation of APs. This can be achieved by confocal microscopy and/or Western blot assays following autophagy marker proteins (ATG8 for example) coupled to fluorescent tags; or by labeling APs with acidotropic dyes (for example, monodansylcadaverine (MDC), monodansylpentane (MDH), and acridine orange) but with a lower specificity and sensitivity [91]. The use of concanamycin A, a molecule which results in the inhibition of vacuolar hydrolases, can also be used to visualize the accumulation of ABs within the vacuole. Using these techniques, autophagy was shown to exist at a basal level in favorable environmental conditions; in turn, the rate of AP formation, their number and overall autophagy activity, is significantly increased by a plethora of environmental stresses [46,85,92] (Figure 2).

The induction of autophagy in response to environmental or developmental cues is finely regulated by multiple mechanisms. Notably, several studies have shown that the expression of *ATG* genes is modified during plant development with an upregulation during senescence [92], and upon exposure to various autophagy-inducing stresses (including nitrogen and carbon deficiency [93,94,95,96], osmotic stresses [52,94,95,97,98], heat and cold stresses [53,94,95,98,99], light quality and intensity [97,100], hypoxia [85], biotic stress [93], and viruses infection [77,78,84,98,99,101]). Transcriptional regulation of autophagy involves WRKY, one of the largest family of TFs in plants [102], as silencing of *WRKY33a* and *WRKY33b* in tomato during heat stress was found to cause a decrease in *ATG* genes expression and a reduction in AP formation [103]. Besides gene regulation, post-translation modifications are also critical for the control of autophagy activity. When growing conditions are optimal, the ATG1 initiation complex is phosphorylated, in particular by the evolutionary conserved complex TARGET OF RAPAMYCIN (TOR), which inhibits the initiation of autophagy [104] (Figure 1). Additionally, the heterotrimeric SNF1-RELATED PROTEIN KINASE 1 (SnRK1) complex also participates in autophagy induction notably by repressing TOR inhibition upon nutrient starvation [2]. Although current knowledge of the mechanisms by which autophagy is instructed and regulated is mostly based on research on ATG genes/proteins, increasing evidence presented below now suggest that membrane lipids are also essential actors of the autophagy pathway.

### 3.1. Availability of Lipids Is Key to AP Membrane Formation and Sustained Rates of Autophagy Flux

In addition to ATG proteins, autophagy is also dependent on lipids which, at the very least, provide the building blocks required for the sustained formation of autophagic membranes. In fact, a considerable amount of lipids is required to support phagophore expansion and AP formation, whether these lipids come from pre-existing organelles or are produced at the phagophore membrane. In mammals, upon nutrient starvation-induced autophagy, the number of APs present in one cell at a given time can go up to about 100 structures per cell [105,106]. In plants root cells, upon nutrient starvation during a few hours, the number of autophagic structures observable inside of the vacuole (in plants concomitantly treated with concanamycin A to accumulate ABs) can go up to more than 100 APs per cell as well (Figure 2).

**Figure 3:**
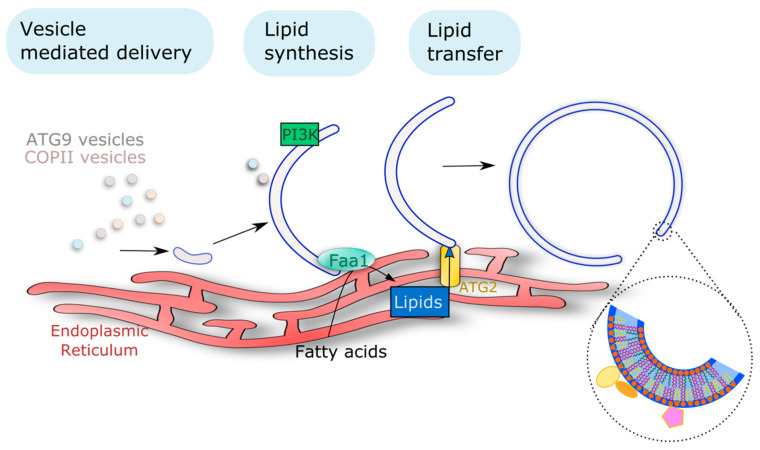
Multiple membrane sources support phagophore assembly and expansion as reported in yeast and mammals. Different types of endomembrane vesicles (including ATG9 vesicles but also endosomes or COPII vesicles) have been proposed as membrane seeds for the nucleation of the initial structure. Lipid transfer proteins (including ATG2 as shown in yeast and mammals) mediate the expansion of the phagophore by promoting the exchange of lipids, actively synthesized in the ER, to the growing phagophore at membrane contact sites. Additionally, on-site lipid synthesis also participates in phagophore expansion, with the example of localized PI3P synthesis (conserved from yeast to plants and mammals). The insert highlights the requirement of a specific lipid composition to support the phagophore architecture and its activity, notably by mediating the recruitment of ATG proteins represented by the color forms. PI3K, Phosphatidylinositol-3-kinase; Faa1, Fatty acid activation 1.

Knowing the size and number of APs present per cell in mammals, Melia and colleagues, made a clever estimation of the number of lipids that would be mobilized in order to create all the membranes required for AP formation in starvation conditions [107]. Their estimation was that, in these conditions, one cell would need the mobilization of 100,000,000-plus lipids to support AP growth. This means that a considerable amount of lipids needs to be remobilized (or synthesized) to ensure the autophagic process. The fact that APs are mostly devoid of transmembrane proteins has made it difficult to trace the origin of the phagophore lipid/membrane. Recent advances in the field of autophagy, mostly based on work in yeast and mammals, have allowed the proposal of the main mechanisms by which lipids are remobilized to the formation of these autophagic structures: vesicle mediated delivery, protein mediated transport of lipids, and on-site lipid synthesis (Figure 3). Such mechanisms and how they affect the autophagic pathway are described below.

#### 3.1.1. Vesicle Mediated Delivery

ATG9, the sole transmembrane protein among ATGs, cycles between different vesicular pools and is required at the PAS for the formation of APs. In yeast, Atg9 exists predominantly in 30–60 nm diameter cytoplasmic vesicles (called Atg9 vesicles) derived from the Golgi apparatus [108]. Upon autophagy activation, Atg9 vesicles are directed towards the site of AP formation and their membranes are thought to serve as seeds for the nucleation of the initial autophagic membrane together with additional membrane sources [109]. In addition to provide an initial platform for the formation of autophagic structures, ATG9 vesicles have important roles in other aspects of the process. A recent outstanding study preformed in yeast showed that ATG9 vesicles can be used as substrates for the PI3K complex for in situ PI3P production [110]. The same study showed that this in situ PI3P production mediated the recruitment of downstream PI3P-effectors and the conjugation machineries which allowed for the ATG8 lipidation to occur in ATG9 vesicles [110]. In this manner, this study showed that ATG9 vesicles constitute a platform for the recruitment of autophagic machinery and thus are critical for the nucleation and expansion of an initial autophagic membrane in yeast. A difference between yeast and mammals is that in the latter, Atg9 vesicles do not fuse and coalesce with autophagic structures, they only transiently interact with them [111]. However, in both organisms Atg9 vesicles mediate the transport of essential elements required for AP formation. In fact, it was shown that Atg9 vesicles serve as a vehicle for the delivery of a class III phosphatidylinositol-4-kinase PI4KIIIβ to the autophagy initiation membrane in mammals [112]. The trafficking of yeast Atg9 vesicles and the subsequent AP formation depends on the activity of this PI4K, indicating a tight connection between Atg9 vesicles and the phosphoinositide PI4P.

Plant ATG9 also reside in vesicles that relocalize to AP formation site upon autophagy induction [24]. However, plant ATG9 seems to have distinct functions from its counterparts in mammals or yeast. In yeast and mammals, mutation in ATG9 results in an inability to form APs [109,113]. In contrast, ATG9 plant homolog is actively implicated in autophagy but not mandatory, as it was shown that the *atg9* mutant shows a decrease, but not a total block in the autophagic flux [114]. ATG9 was further described as required for the formation of APs from ER membranes [24]. In this same study, authors found that *atg9* mutants are still able to form GFP-ATG8a labeled structures with direct connection with the ER. However, these structures are abnormally large and misshaped [24]. Taken together, this information strongly suggests that ATG9 is not the main source of lipids for AP formation, and that, in plants, ATG9 vesicles may have a role in later stages of the process. Notably a recent study determined the structure of Arabidopsis ATG9 (ATG9) by cryo-EM, and showed a membrane-bound core region consisting of six transmembrane domains flanked by disordered N- and C-terminal cytoplasmic regions [115]. It was also found that, like its yeast counterpart, plant ATG9 self-interacts, and upon trimer formation, the C-terminal folds into a more stable conformation, which could in turn provide a scaffold for subsequent protein interactions [115]. Finally, both yeast and mammalian ATG9 were recently found to be capable of translocating phospholipids between membrane leaflets in vitro [116]. This means that ATG9 is locally capable of modifying the leaflet lipid composition of autophagic membranes, indicating a possible role of this protein in the remodeling/shaping of autophagy membranes. Whether this function is conserved in plant ATG9 remains to be tested.

Other types of vesicles have also been implicated in the formation of autophagic structures in yeast and mammals. The endoplasmic reticulum–Golgi intermediate compartment (ERGIC)-derived COPII vesicles have been proposed as alternative/additional membrane seeds implicated in the nucleation of the phagophore as they have been shown to relocalize to the site of AP formation [20,21,22]. COPII vesicles play an important role in the transport of proteins and membranes from the ER to the Golgi apparatus. For example, yeast mutants deficient in COPII vesicle formation cannot form APs [117]. Furthermore, it was later shown, in yeast cells, that COPII vesicles mediate the transport of a ER-localized protein implicated in AP formation (mutants displayed less APs) to the site of AP biogenesis [118]. Moreover, it has been proposed that COPII vesicles become part of AP membranes upon the transport of proteins to APs in both yeast and mammalian systems [19,20]. Another type of vesicles implicated in autophagy are endosomes. In mammals, ATG9 has been shown to traffic between the PM and the trans-Golgi network (TGN) via endosomes that eventually coalesce to provide an initial membrane for autophagy [18,119,120]. This highlights the potential importance of multiple organelles in the formation of autophagic structures. In plants, very little is known at this time about the contribution of intracellular vesicles to the delivery of lipids for AP formation although endosomal compartments have been proposed to participate in autophagy suggesting a potential cross-talk between endocytosis and AP formation [121].

#### 3.1.2. Action of Lipid Transfer Proteins

As mentioned previously, multiple organelles, especially the ER, have been shown to contribute to the formation of autophagic membranes. The ER is the central organelle where most of the lipid synthesis occurs [122], and as such, represents a potential critical source of lipids for the formation of APs. In fact, besides vesicle-mediated delivery, lipids required for AP formation can also be transferred to the autophagic structures directly by lipid transfer proteins (LPTs, Figure 3) [119]. These proteins benefit from the numerous membrane tethers that we have described above to transfer lipids from one organelle to phagophores. In yeast, the ATG18-ATG2-ATG9 complex has been shown to allow the establishment of contact sites between the ER and the edges of the growing phagophore [23]. Interestingly, mammalian ATG2 was found to localize to mitochondria-associated ER membranes to allow phagophore progression [120] as well as displaying a dual localization on both autophagic membranes and lipid droplets (LDs) [123]. These results indicate that the ATG18-ATG2-ATG9 complex is able to tether different membranes to that of the growing phagophore, presumably to use them as lipid sources for phagophore expansion [124,125]. Besides its role in creating membrane tethers between different organelles, a series of recent studies determined another role for ATG2 in AP biogenesis. ATG2 was recently characterized as having lipid transfer capabilities in mammals [126] and in *Saccharomyces pombe* [127]. In *S. pombe*, Atg2 displays a hydrophobic cavity capable of accommodating phospholipid acyl chains that was found to act as a bridge allowing the transfer of lipids [127]. In this manner, mammalian ATG2 was found to bind up to 20 different lipid molecules at a given time, and to be capable of lipid transfer into liposomes *in vitro* [128]. This places ATG2 as a major actor in the expansion of phagophores through its lipid transfer capacities, and highlights the importance of such mechanisms in the autophagic process.

Similarly, GRAMD1A (GRAM DOMAIN-CONTAINING 1A) is another LTP characterized as implicated in AP formation. GRAMD1A is a cholesterol transfer protein that was seen to relocalize to the site of AP formation in a PI3P-dependent manner in mammalian cells. Similar to ATG2, the inhibition of GRAMD1A leads to an absence of AP formation, indicating its crucial role in the process [129]. Finally, the human VPS13A (VACUOLAR PROTEIN SORTING 13 HOMOLOG A) and VPS13C (VACUOLAR PROTEIN SORTING 13 HOMOLOG C) proteins were also described as LTPs responsible of lipid transfer between the ER and other organelles [130]. Interestingly, VPS13A downregulation leads to an impaired autophagic flux [131]. This could be explained by an inability to form correct autophagic structures due to the absence of the lipid transfer activities of VPS13A as the authors observed a reduced rate of ATG8 lipidation in VPS13A knockdown cells [131].

ACYL-COENZYME A BINDING PROTEINS (ACBP), which bind and carry acyl-CoA esters, are interesting additional protein players in the connection between autophagy and lipids. In fact, the injection of the ACBP protein in mice induces the inhibition of autophagy, lipogenesis, reduces blood sugar levels, and stimulates appetite resulting in weight gain [132]. In yeast, the deletion of Acb1 (*Δacb1*) inhibits autophagy during chronological aging but is also responsible for the inhibition of the food seek strategy called sporulation [133]. Conversely, the single *acpb-1* mutation or quadruple *acbp1;3;4;6* mutation induces autophagy in the nematode *Caenorhabditis elegans* but reduced the uptake of bacteria and the pharyngeal pumping [133]. Finally, in *Drosophila melanogaster*, the mutation of the *Anorexia* (*Anox*) gene, encoding an ACBP with an ankyrin repeat domain, reduces the feeding and chewing activities of the fruit fly [134]. For its role in controlling appetite in several eukaryotic organisms, ACBP has recently been named as “hunger factor” [133]. In Arabidopsis, Xiao and colleagues showed that overexpression of ACBP3, a PE and phosphatidylcholine (PC) interacting protein, causes an acceleration of senescence coupled with an enhanced degradation of GFP-ATG8e suggesting that ACBP3 may regulate leaf senescence mediated by autophagy [135], although its exact function in this process remains elusive at this time.

The identification of lipid carrier proteins greatly improved our understanding of the mechanisms leading to phagophore expansion in yeast or mammals. More studies are nevertheless needed to fully understand these lipid-transfer models, such as determining the lipid preferences of these proteins as to having a better image of their contribution to the lipid composition of phagophores. Moreover, in plants direct implication of LTPs in autophagy has not been described thus far. Plant ATG2 has been identified and is strictly required for autophagy; furthermore, ATG2 was implicated in maintaining ROS homeostasis [136], and chlorophyll degradation upon abiotic stress [60]. The molecular function of ATG2 in plant autophagy remains elusive, but, interestingly, plant *atg2* mutants are capable of forming small-size ATG8-positive structures which presumably correspond to phagophores or immature autophagic structures unable to fuse with the vacuole and thus accumulate in the cytosol [137]. This raises the question of the conservation of ATG2 lipid transfer function in plants which should be an exciting focus for future research.

#### 3.1.3. On Site Lipid Synthesis

In addition to vesicular trafficking and lipid transfer, lipids required for AP formation can also be synthesized directly on autophagic membranes. For example, and as mentioned above, the localized production of PI3P by the PI3K complex at the phagophore structure is a prerequisite for AP formation by mediating the recruitment of several ATG proteins that bind to this lipid [138]. This PI3P localized production is a conserved hallmark of autophagy. However, how PI3P is distributed within the autophagic membranes differs between organisms. In yeast, PI3P is present in both leaflets of the AP membrane, with a slight accumulation in the luminal leaflet whereas in mammals, PI3P was found exclusively in the cytosolic leaflet of APs [139]. The fact that PI3P is present in one leaflet more than another might play a role in the recruitment of downstream effectors in specific parts of the autophagic structures. Besides the localized PI3P production, other lipid synthesizing enzymes are also recruited to the autophagic structures, supposedly for lipid production to promote AP formation. Both phospholipases D (PLD) and the PI4 Kinase enzymes colocalize at autophagic structures in mammalian cells, even though their exact functions in autophagy are still to be determined [112,140]. Furthermore, the lipid PI4P is unevenly distributed in both leaflets of phagophore membranes from yeast and mammals, suggesting that the action of PI4K is tightly regulated in space [141]. Another example is the PHOSPHATIDYLINOSITOL SYNTHASE (PIS1) enzyme, which was shown to accumulate at the site of phagophore nucleation on ER subdomains of mammalian cells, and to colocalize with components of the initiation machinery [123]. Thus, PI seems to function in the recruitment of the initiation complex in a step preceding the localized synthesis of PI3P [123]. PIS1 was later found to colocalize with the ER-phagophore contact protein VACUOLE MEMBRANE PROTEIN 1 (VMP1) and with the choline/ethanolamine phosphotransferase enzyme [142,143]. Thus, different phospholipid synthesizing enzymes have been shown to localize to sites where the AP and the ER make contact, probably to support the growth of the structure. Other lipid synthesizing enzymes have been shown to act directly at autophagic structures. For instance, the yeast acyl-CoA synthetase Faa1 (Fatty acid activation1) relocalizes to autophagic structures and was shown to activate fatty acids that will later be channeled for contributing to the growth of the phagophore ([144]; Figure 3). Moreover, mammalian PHOSPHATE CYTIDYLYLTRANSFERASE 1 (PCYT1A), which synthesizes PC, was found to be required for AP formation, suggesting that PC is required for autophagy; and furthermore, authors showed that newly synthesized PC was incorporated into APs [145] and thus contributes to the bulk of lipids composing autophagic membranes.

In plants, much less is known concerning the lipid synthesis required for the formation of APs. However, the PI3K complex was shown to co-localize with APs and to ABs within the vacuole. This suggests that the complex is indeed recruited to autophagic structures to produce PI3P and allow autophagy progression [28]. Furthermore, the fact that the PI3K complex stays to autophagic structures up until their vacuolar degradation could mean that PI3P is required for multiple steps in the process [28]. Another possibility could be that the complex is degraded through the autophagic pathway as a way to finely regulate autophagic activity, as it has been proposed for the initiation complex [29].

Taken together, multiple lipid-mobilization mechanisms have been implicated in the formation of autophagic structures, whether it is to support the flux of lipids required for the growth of the structure, or to modulate the specific lipid composition required for the progression AP formation. These three mechanisms (i.e., vesicle mediated delivery, direct lipid transfer, and on-site synthesis, see Figure 3) highlight the fact that a plethora of lipids need to be remobilized for the AP formation, and any de-regulation in these mechanisms hampers the overall autophagic process. However, not all lipids contribute in the same manner to this process. As mentioned above, some lipids are utilized as building blocks for the structure, while others allow for the recruitment of downstream effectors. This emphasizes the high level of regulation that governs these mechanisms. In plants, how lipids are mobilized towards AP formation stays elusive and should be the focus of future research to unravel this critical aspect of autophagy.

### 3.2. Lipids Are Critical Components of the Structure and Identity of the Autophagy Membranes

#### 3.2.1. Lipids Control the Architecture and Functions of Biological Membranes

Besides their role as building blocks to support the formation of AP membranes, lipids have recently emerged as key components of the autophagy machinery in research mostly performed on yeast and mammals. In fact, lipids are not passive constituents of biological membranes; instead, their specific biochemical and biophysical properties determine critical features of membranes [146]. The chemical structure of lipids, such as the size of their polar head and the unsaturation degree of their acyl chains have a great impact on their shape. For example, lipids with a small polar head but highly unsaturated acyl chains will display a conical shape; and on the opposite side, lipids with big polar heads and saturated acyl chains exhibit an inversed cone shape [147]. Hence, when these lipids are incorporated and accumulate into membranes, they are able to create curvatures within these membranes. Furthermore, lipids with saturated acyl chains pack tightly together within membranes, while lipids with highly unsaturated acyl chains do the opposite. Thus, lipids with highly unsaturated acyl chains can create packing defects in membranes that can in turn be utilized by membrane anchored proteins for their incorporation [146]. In this manner, lipid geometry (i.e., shape) determines critical features such as membrane curvature and the lipid density within each membrane. In addition to their structural roles, lipids also contribute fundamentally to membrane activities by mediating the recruitment of specific proteins with lipid-binding domains or through electrostatic interactions. The nature, quantity, and distribution of lipids within membranes thus define a unique identity for each biological membrane, dictating their properties such as curvature and electrostatics, and attracting selected set of proteins that support membrane functions. As in any other membrane compartment, the specific lipid composition of AP membranes is thus likely to provide critical input for their formation and activity, as well as their global architecture. Yet, little information is available regarding the molecular functions of lipids in autophagy and the exact composition of autophagic structures has remained elusive in all organisms. Nevertheless, it was recently reported that, in yeast, Atg8-containing membranes (that could represent phagophores, APs, and/or ABs within the vacuole) have a phospholipid composition of 38% PC, 37% phosphatidylinositol (PI), 19% PE, 3% phosphatidylserine (PS), and 3% phosphatidic acid (PA) [144]. Compared to other endomembranes, ATG8-labeled membranes thus display an enrichment in PI, the precursor of PI3P, which is known to regulate different steps of the autophagic process. Recent reports have shown that PC is synthesized de novo and incorporated into autophagic structures [145] which is in coherence with PC being found in high proportion in Atg8-labeled membranes (38% of phospholipids, [144]). Likewise, the considerable amount of PE compared to other membranes makes sense given the importance of this lipid in AP formation as described below.

In plants the nature, dynamics, and functions of lipids in autophagy structures are still largely unknown. In that context, the following part of the review will present the known roles of lipids in the formation of autophagy membranes with a focus on recent research performed in yeast and mammals. Note that fundamental differences exist in the mechanism of autophagy across eukaryotes and, likewise, the lipid composition of autophagic structures and their function in AP formation may differ from one organism to another. While caution must be taken in extrapolating these discoveries to plants, these recent findings certainly prompt consideration of the potential contribution of lipids as critical actors of the plant autophagy pathway.

#### 3.2.2. Lipid/Protein Binding: Lipids Mediate the Transient Association of ATG Proteins with the Phagophore

**Figure 4:**
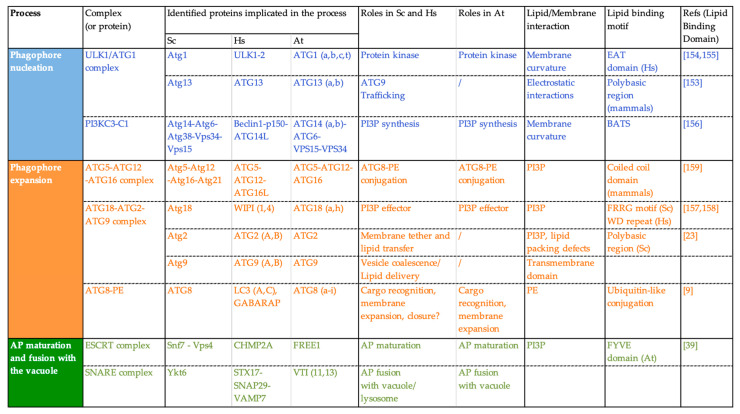
Key proteins of the autophagy machinery, function and interaction with lipids/membranes in *Saccharomyces cerevisiae* (Sc), *Homo sapiens* (Hs), and *Arabidopsis thaliana* (At).

Upon autophagy induction ATG proteins are very rapidly recruited from the cytosol, where they normally reside, to the lipo-proteic PAS structure to nucleate the initial autophagic membrane. The recruitment of ATG proteins relies on protein–protein interactions but also on the binding of ATG proteins to lipids or to specific lipid-mediated membrane properties. In fact, several ATG proteins—from different ATG complexes—contain either lipid-binding motifs or positively charged regions that allow them to bind lipids through electrostatic interactions [23,148,149]. In addition, other ATG proteins are able to sense membrane features such as membrane curvature or lipid packing defects (Figure 4; note that this list is not exhaustive, examples have been chosen to illustrate the different steps of AP formation in which interactions with lipids are important).

For example, yeast Atg1 displays an early autophagy targeting (EAT) domain that is able to sense membrane curvature with a preference for highly curved membranes [150,151]. In the same manner, mammalian ATG14, a member of the PI3KC complex in mammals, is able to recognize membrane curvature through its Barkor/Atg14(L) Autophagosome Targeting Sequence (BATS) domain [152]. These membrane-sensing capacities of ATG proteins allow them to relocalize specifically at the highly curved edges of the phagophore and thus contribute to the high spatiotemporal regulation that governs the AP biogenesis process. The recruitment of the PI3KC complex to membranes with high curvature allows for the localized production of PI3P required for the progression of AP formation. In turn, PI3P allows for the recruitment of what are called “PI3P-effectors” such as the ATG18 proteins. Yeast Atg18 is a seven bladed β-propeller protein and binds PI3P through an FRRG motif [153]. The Atg18 protein partner Atg2, was found to bind to PI3P containing liposomes and this interaction was increased in ergosterol-containing liposomes (ergosterol creates lipid packing defects in membranes) suggesting that Atg2 is also capable of sensing packing defects [23]. In mammals, ATG18 proteins bind PI3P through two distinct recognition sites: the FRRG motif as it occurs in yeast, and through the WD-repeat domain Interacting with phosphoinositides [148]. It has been further reported that mammalian ATG16L was able to bind phosphoinisitides, notably PI3P, through its coiled-coil domain (CCD) and the deletion of this domain resulted in an incapability to lipidate ATG8 [149]. In mammals, another protein implicated in the conjugation of ATG8 to PE, Atg3, contains an amino-terminal amphiphatic helix and was found to promote ATG8 lipidation in vitro in liposomes with a high curvature [154].

Thus, it would seem that ATG8 lipidation, as well as other hallmarks of autophagy, is spatially regulated by a wide range of lipid/protein interactions, many of them involving PI3P but also mediated by membrane features, like membrane curvature or packing defects. In plants, PI3P is also critical for autophagy, as mutants for genes part of this complex display a hampered AP formation [52]. The autophagy specific PI3K complex and plant PI3P-effectors, such as SH3P2 (SH3 DOMAIN-CONTAINING PROTEIN 2) or ATG18, have also been characterized and evidence have been provided for the importance of PI3P in plant autophagy [24,28,155]. For example, the PI3P-binding SH3P2 protein was found to promote PI3K foci formation and to interact with ATG8, and was thus proposed as playing important roles in AP progression [156]. Moreover, the FREE1 protein that we mentioned above in this review, was found to interact with SH3P2 and ATG6, a member of the PI3K complex [39]. Finally, ATG5 was found to localize specifically to the rim of phagophore structures, suggesting that some specific membranes features such as curvature dictate the localization of the ATG5-ATG12 complex as well [13].

Another phosphoinositide important for the formation of APs is PI4P. In both mammals and yeast, PI4P plays important roles in the trafficking of ATG9 vesicles [112,157]. Both PI3P and PI4P have been shown to mediate multiple steps in the autophagic pathway. For example, a clearance of PI3P from APs is required for autophagy, as the loss of function of the myotybularin PI3P phosphatase Ymr1 resulted in a continuous association of ATG proteins with completed APs which failed to fuse with vacuole in yeast [158]. This phenomenon has also been observed in *C. elegans* [159]. However, PI3P has also a role in yeast AP maturation as it facilitates the recruitment and stabilization of specific proteins required for its fusion [160,161] highlighting that these events are highly regulated in a spatiotemporal manner. Moreover, a deficiency of the yeast PHOSPHATIDYLINOSITOL-3-PHOSPHATASE SAC1, responsible for cleaving the phosphate moiety of PI4P, lead to an abnormal accumulation of PI4P in APs and resulted in a failure to recruit the SNARE complexes required for AP fusion with the vacuole [74]. Another recent publication also identified the SAC1 interactor TMEM39A (TRANSMEMBRANE PROTEIN 39A) which promotes the intracellular trafficking of SAC1 to regulate the spatial distribution and levels of PI4P [155]. These results suggest that similarly to PI3P, the PI4P pool at autophagic structures is under highly regulated turnover to support the proper functioning of the autophagic process.

Interestingly, both of PI3P and PI4P display asymmetrical distributions on autophagic membranes from yeast and mammals [139,141]. The geometry of both these lipids (with a polar head larger than its acyl chains) confers them an inverted cone shape. When lipids with an inverted cone shape cluster within membranes, they can deform membranes and create curvature [147]. Thus, the asymmetrical distribution of PI3P or PI4P within phagophores membranes could contribute to its highly curved architecture. Another critical lipid for autophagy is PE. Conjugation of the ATG8 protein to PE allows for its incorporation into autophagic membranes. The incorporation of ATG8-PE to the phagophore is thought to support the expansion of the growing structure [162]. Furthermore, the incorporation of ATG8-PE contributes to the membrane curvature of the phagophore in yeast cells [163]. Finally, other phosphoinositides such as phosphatidylinositol 4,5-biphosphate, PI(4,5)P_2_, and phosphatidylinositol 3,5-biphosphate, PI(3,5)P_2_ have been shown to recruit proteins implicated in autophagy. PI(4,5)P_2_ was found to mediate the recruitment of the endomembrane trafficking regulator SNX18 (SORTING NEXIN 18) in human and drosophila cells, which further recruited ATG16L to perform ATG8 lipidation but also contributes to the trafficking of ATG9 vesicles [164,165]. The implication of PI(3,5)P_2_ in autophagy has also been reported although the exact molecular mechanism remains elusive. A reduced phosphoinositol-3,5-kinase Fab1 activity lead to an impairment in AP/lysosome fusion [166]. Nevertheless, an impairment of a PHOSPHATIDYLINOSITOL POLYPHOSPHATE 5-PHOSPHATASE TYPE IV (INPP5E) also lead to this same phenotype [167]. This indicates that the modulation of PI(3,5)P_2_ levels on autophagic structures is tightly regulated to allow proper fusion with lysosomes.

At present, several lipids have been reported to have a critical role in the progression of autophagy and their function as binding modules for the recruitment of ATG proteins has been proposed. However, information about their quantity, dynamics, and repartition within the membranes of APs remains scarce. How these impacts on the shaping and ultrastructure of the phagophore thus remain a fundamental open question that future research will need to address to resolve the puzzled mechanisms of AP formation.

### 3.3. Lipids Are Messengers for Autophagy Induction

In addition to mediating ATG protein interaction with the phagophore, some lipids also play key roles in the regulation of autophagy. PA was shown to modulate autophagy in mammalian cells. The PA-synthesizing enzyme PLD1 relocalizes to autophagic structures and its absence greatly impairs autophagic activity [168]. Moreover, the PA-binding protein HCLS1-BINDING PROTEIN 3 (HS1BP3) has been shown to negatively affect autophagy by modulating the activity of PLD1 and PA levels [140]. These results suggest that the synthesis of PA is required at the site of AP formation. Although recent evidence suggest that PA is present at low concentration on the membranes of autophagy structures in yeast [144], its function in the structure or activities of autophagy membrane remains largely unknown. Besides its role as a structural component, PA is also known to regulate autophagy in mammals by stimulating the mTOR pathway which suppresses autophagy [169]. mTOR is a master regulator capable of transducing nutrient signals and to regulate several cellular functions, and one of mTOR’s functions is to inhibit the autophagy pathway [169]. This implicates two distinct functions of PA concerning autophagy. One possible explanation is that different pools of PA are responsible for these distinct functions, one pool of PA might be incorporated into APs, while the other pool could be responsible for interacting with mTOR to suppress autophagic activity. Further studies await to reveal the molecular role of PA in the fine-tuning of autophagy activity.

Another class of lipids implicated in the regulation of autophagy are sphingolipids. Two sphingolipids have been shown to play important roles in autophagy regulation: ceramide and sphigosine-1-phosphate (S1P). Contrary to PA, ceramides inhibition of Akt, a mTOR regulator, results in mTOR inactivation and thus in autophagy activation [170]. The activity of the S1P synthesizing enzyme, the SPHINGOSINE KINASE (SPHK), was shown to also induce autophagy in mammalian cells, through an Akt-independent deregulation of the mTOR pathway [171]. Multiple fatty acids have also been shown to regulate autophagy. The highly unsaturated docosahexaenoic acid (DHA) (22 carbons and six unsaturations, 22:6) was found to induce autophagy in human cancer cell lines by AMP-activated protein kinase (AMPK)-mediated inhibition of mTOR [172]. Interestingly, it was also found that palmitate (16:0) and oleate (18:1) induced autophagy in mammalian systems, although through distinct mechanisms. Palmitate supplementation activated autophagy via the canonical PI3K-induced autophagy pathway [173]. Oleate, on the other hand, was able to induce autophagy even in cells defective for the PI3K complex, indicating that oleate activates a non-canonical autophagic pathway [173]. Finally, a pharmacological inhibition of cholesterol in mammalian cells lead to an activation of autophagy due to the inactivation of the mTOR pathway [174].

Throughout this section, we have seen that lipids are key actors in the autophagic process. They not only contribute to defining the expansion and particular shape of autophagic structures, but also serve as anchors for the recruitment of many membrane-associated proteins that drive this process. In plants, little is known about the lipids that are present on autophagic structures as well as their functions in AP formation. Different types of stress can induce autophagy, with a specific autophagic response for each of them [175]. It is likely that different types of stress induce the formation of distinct populations of APs, whose lipid composition might as well vary depending on the stress. We know that autophagy is critical for plants’ response and adaptation to stressful environments, yet little is known about the lipid composition and functions in the formation of autophagic structures upon each stress and/or on the type of cargo to be engulfed. Future studies are required to unravel the nature, dynamics, and functions of lipids in plant APs. These should provide valuable insights into the understanding of how lipids functional contribute to the autophagic-mediated acclimation of plants to environmental stresses.

## 4. Autophagy Rewires Cellular Activities to Mediate Plant Adaptation to Stress: The Complex Relationship between Autophagy and Lipid Metabolism

**Figure 5:**
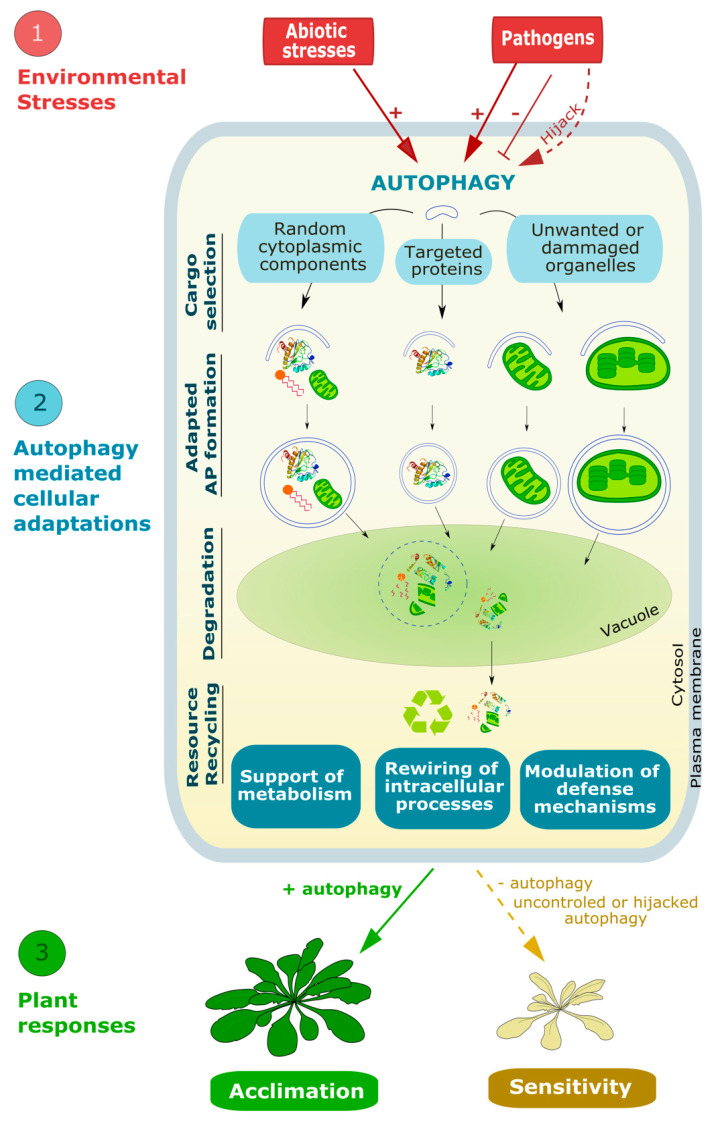
Schematic representation of autophagy-mediated mechanisms leading to plant acclimation to environmental stresses. Different types of stress can either induce, inhibit, or hijack autophagy. Upon autophagy induction, and depending on the type of stress, growing APs target and engulf selected components on the cells thus recycling nutrients, clearing cells from unwanted organelles and redirecting plant metabolism to respond to the environmental conditions and promote plant tolerance. On the other hand, when the autophagic process is inhibited, or hijacked notably by viral or bacterial pathogens, plants are unable to adapt to these stresses, thus resulting in plants’ sensitivity and ultimately death.

As described in the previous section, reverse genetics approaches have highlighted the importance of autophagy for plant physiology thereby unravelling the mostly pro-tolerance role of autophagy in plants acclimation to environmental stresses. How does autophagy impact and regulate such a variety of aspects in plant physiology? Recent advances show that, by degrading cell components (in bulk or in a selective manner), autophagy can serve as a quality control mechanism for the removal of damaged macromolecules and organelles, and by this redirect discrete cell activities to cope with harsh environmental conditions (Figure 5).

Notably, non-selective autophagy towards random cargo or proteins is involved in nitrogen recycling for the remobilization of nutrients from source leaves to sinks which is particularly enhanced during leaf senescence or in response to nitrogen starvation [176]. Studies using ^15^N isotope labelling and tracing showed that nitrogen fluxes from rosette leaves to seeds were strongly decreased in Arabidopsis autophagy mutants [4] and similar conclusions were reached for maize [5] and rice [177]. In addition to nitrogen, autophagy was shown to also recycle iron and sulfur [178,179], and certainly other elements like manganese and zinc. In addition to the random non-selective autophagy, APs can also selectively target specific proteins to modulate key plant metabolic pathways or intracellular processes. For example, autophagy participates in the degradation of TRYPTOPHAN-RICH SENSORY PROTEIN (TSPO) [180], a stress inducible protein [181,182]. TSPO was found to interact with the aquaporin PLASMA MEMBRANE INTRINSIC PROTEIN 2;7 (PIP2;7) within the early secretory pathway and to downregulate its abundance in the cell [183]. It was proposed that the heterocomplex is delivered in the vacuole by autophagy, thereby reducing the abundance of PIP2;7 at the PM and protecting the cell from water deficit [183]. Another example of autophagy-mediated regulation and adaptation to stress by means of protein degradation is the control of the level of the protein ARGONAUTE1 (AGO1) in the context of viral infection. AGO1 is a component of the RNA-induced silencing multi-protein complex (RISC) associating with small interfering RNA (siRNA) thus allowing gene silencing in plants. Derrien and colleagues showed that the viral suppressor of RNA silencing P0 protein encode by the Polerovirus triggers the degradation of AGO1 by autophagy [184], modulating gene silencing and allowing viral replication. Besides specific proteins, selective autophagy also modulate the adaptation of plants to environmental stresses by degrading unwanted, damaged, or supernumerary organelles including mitochondria [46], peroxisomes [185], non-functional 26S proteasome [186], parts of the ER [187,188], and the nucleus [189]. Last of all, chlorophagy, i.e., the selective degradation of a chloroplast, is a peculiarity of photosynthetic organisms compared to mammals and yeasts [6,190,191]. By degrading these aforementioned cellular ‘machines’, autophagy participates in the redirection of cell metabolism and/or activities. For example, Üstün and colleagues showed that *Pseudomonas syringae pv tomato* can activate autophagy with the type III effector (T3E) and stimulate autophagic removal of proteasomes via proteophagy to promote bacterial proliferation [192]. Further, the recycling of chloroplast proteins during senescence is an important event for the maintenance of homeostasis of the plant. This is made possible in particular by the transfer of proteins, such as the RIBULOSE-1,5-BIPHOSPHATE CARBOXYLASE-OXYGENASE (RUBISCO), from the stroma via spherical double membrane structures called Rubisco containing bodies (RCBs) using the autophagy pathway [6,190,191]. In addition, autophagy degradation also instructs plant physiology by participating in the regulation of lipid metabolism, as presented and discussed below.

### 4.1. Autophagy Regulates Lipid Metabolism

As mentioned above, studies performed by Ishida and Izumi’s groups provided evidence that chloroplasts are degraded through both macro- and micro-autophagy pathways [193]. This demonstration is in line with the role of autophagy in nitrogen remobilization throughout the plant as chloroplasts represent the main nitrogen source in leaves. Microscopy experiments showed that RCB are extruded from chloroplasts and taken in charge by macro-autophagy for delivery to the vacuole [194]. Consistent with their names, RCB contain RUBISCO and additional stromal proteins, like the chloroplastic GLUTAMINE SYNTHETASE 2, GS2, for example, but no chlorophyll or photosynthesis antenna proteins. Thylakoid proteins were found in vesicles originating from the chloroplast, the latter of which contain ATI1 (ATG8-INTERACTING PROTEIN 1) proteins bound to ATG8f [195,196]. The release of stromal proteins from chloroplast through RCB, and of thylakoid material through these so-called ATI-PS vesicles, may contribute to chloroplast shrinkage under stressful conditions like carbon starvation and salt stress, respectively [190]. Chlorophagy that consists in the degradation of entire chloroplast in the vacuole was further demonstrated. The engulfment of UV-damaged chloroplast by invagination of the tonoplast in Arabidopsis was imaged. Such micro-autophagy of damaged chloroplast is dependent on ATG8 and does not occur in the *atg5* mutant [193]. ATG8-labelled cup-shape structures were detected in between the damaged chloroplasts and the tonoplast. Thus, involvements of proteins from the macro-autophagy core machinery are likely needed in the micro-autophagy process. Altogether, these studies demonstrated the occurrence of chlorophagy in dark-stressed and UV-treated plants. However, the proteomic, lipidomic, and metabolomic approaches carried out in both Arabidopsis [191] and maize [197] revealed lower chloroplast protein and lipid levels in autophagy-deficient mutants, suggesting that autophagy is non-essential for chloroplast degradation during leaf senescence, or at least that alternative pathways can efficiently take in charge chloroplast decay during aging.

As exemplified in the two latter studies, steady-state levels of chloroplastic lipids, such as monogalactosyldiacylglycerol (MGDG) and digalactosyldiacylglycerol (DGDG), decrease in both Arabidopsis and maize autophagy mutants whereas other structural lipids (phospholipids, sphingolipids) engaged in extra-plastidial membranes over-accumulate in those mutants [191]. Amongst sphingolipids, the glycosylinositol-phosphorylceramides (GIPCs) showed a marked concentration changes in the Arabidopsis *atg5* mutant [191]. Since GIPCs are mainly found in the PM [198] and, to some extent, in the tonoplast [199], these data pinpoint, at least, an unexpected role for autophagy in the homeostasis of the PM. In addition, the observed phospholipid accumulation in *atg5* mutants [191] may be tentatively explained by a defect in ER-phagy [200] that could provoke an increase in ER membrane surface. Proteomics also revealed a constitutive loss of ER homeostasis associated with the onset of the peroxisomal β-oxidation in the Arabidopsis *atg5* mutants [191]. Both ER and peroxisome are known to play a crucial role in lipid metabolism. The decrease in MGDG and DGDG and the higher levels of the β-oxidation enzymes in *atg5* mutants indicate that chloroplast lipids surely fuel β-oxidation in mutants. Again, in line with lipidomic analyses, the perturbations of ER function in *atg5* may result from improper ER-phagy, suggesting that autophagy could be involved in lipid recycling from endomembranes under such circumstances. Using the maize *Zmatg12* mutants, McLoughlin and coworkers further reported on modifications of glycerolipid contents that could support an increase in triacylglycerols (TAG) storage lipids that could be consumed by β-oxidation or mobilized for membrane remodeling [197]. Consistently, the formation of cytoplasmic TAG-containing LDs was also suggested by Havé and colleagues [191], since the PHOSPHOLIPID-DIACYLGLYCEROL ACYL-TRANSFERASE 1 (PDAT1) enzyme, in charge of TAG synthesis at the ER membrane, was more abundant in the Arabidopsis *atg5* mutant. However, TAG contents in adult plants were not large enough to detect any convincing difference in TAG content between *atg5* and WT plants in this study.

Remarkably, the autophagy function in endomembrane recycling was elegantly confirmed recently [201]. Using ^14^C-acetate labelling, the authors performed chase experiments on the Arabidopsis *atg5* and *atg7* mutants and demonstrated that autophagy is a genuine actor of endomembrane lipid turn-over in senescing leaves. Indeed, genetic inactivation of autophagy inhibited fatty acid (FA) mobilization from membranes for TAG synthesis. By contrast, ^14^C labelling in young growing and mature leaves unravelled the participation of basal autophagy in the neo-synthesis of FA and TAG. However, under dark-induced senescence conditions, Fan and coworkers also showed that autophagy had no impact on chloroplast FA synthesis and that the recycling of thylakoid membranes was not modified in autophagy mutants [201]. They thus concluded that inducible macro-autophagy was involved in the degradation of lipids originating from endomembranes, but not in that of chloroplast lipids. In the same work, electron micrographs carried out on dark-stressed seedlings evidenced the degradation of cytoplasmic LD, a phenomenon referred to as lipophagy. Given that this lipophagy phenotype resembled a micro-autophagy process and could be blocked by the *atg2-1* mutation, the authors assumed that LD micro-autophagy requires the macro-autophagy core machinery. More recently, lipidomic analyses on the Arabidopsis *atg5*, *atg7,* and *atg9* autophagy mutants grown under dark conditions confirmed that autophagy affects the homeostasis of multiple lipid families [202]. Once again, the degradation of chloroplast lipids was observed in the *atg* mutants, like in the aforementioned studies. However, unlike in the experiments performed under N or S limitations [191], Barros and colleagues observed an accumulation of plastid-derived TAG and of plastoglobuli in *atg* mutants [202]. However, despite TAG accumulation under dark stress, autophagy mutants contained surprisingly less LDs. This indicates that lipid storage in autophagy mutants is dependent on the nature of the nutritional stress and is mainly promoted by carbon starvation. Nevertheless, irrespective of the growth conditions––whether plants were stressed or unstressed—the β-oxidation process was enhanced in autophagy mutants. This typically shows that lipids are important substrates that sustain respiration and energy production under autophagy deficiency.

### 4.2. Autophagy, Lipids, and Unfolded Protein Response, a Third Player in the Game?

The ER, along with plastid, is one of the main subcellular site of lipid neo-synthesis in plants. Endoplasmic membranes encompass multiple lipid biosynthesis enzymes, likely associated in complexes that can elongate and/or channel long-chain acyl Coenzyme A originating from plastids towards phospholipid, TAG and sphingolipid production [203]. Interestingly, ER is also the gate to the secretory pathway for soluble and transmembrane proteins to be secreted, once they have been co-translationally translocated across/within the membrane, folded and maturated by resident (co-)chaperones and glycosidases [204,205]. Sustaining such an intense metabolic activity while maintaining ER homeostasis is therefore of utmost importance for proper cell functioning. Originally, ER stress has been defined as a proteo-toxic stress characterized by a disequilibrium between the cell demand in secreted proteins to be translated by the organelle and its capacity to do so. In other words, when unfolded/misfolded proteins accumulate within the ER lumen, it is considered that the organelle homeostasis is disrupted. However, it is now also commonly admitted that lipo-toxic stress, resulting from modifications of the ER membranes (namely oxidation or increase in its saturation degree), also represents a genuine ER stress, at least in animal and yeast cells [206].

In eukaryotic cells, a conserved retrograde signalling pathway, so-called the Unfolded Protein Response (UPR), is activated under ER stress conditions. This stereotyped path is launched by the remobilization of ER-localized bZIP TF to nucleus. It has a dual, sequential function: its primary activation aims at restoring organelle homeostasis (pro-adaptive pathway) but, if it fails due to prolonged or acute ER stress, UPR shifts to a pro-death mode to get rid of malfunctioning cells (see [207,208] for reviews on cell death aspects). Pro-survival role of UPR is exerted through several strategies, which relies, for the most part, on bZIP TF-mediated transcriptional reprogramming. For instance, limiting unfolded protein accumulation within lumen can be achieved by increasing the concentration of ER-resident chaperones and additional actors of the ER-Quality Control (ER-QC) system [209]. This eventually enhances ER folding capacity and is thought to be accompanied by a phospholipid neo-synthesis that drives ER membrane expansion, *in fine* augmenting ER volume [210,211]. General translation, as well as ER-dependent translation, can be down-regulated for restricting the load of nascent polypeptides into the ER [212]. Degradation of mRNA associated with ER membranes can also contribute to alleviate unfolded protein burden within the lumen [213]. In addition, unfolded proteins are retro-translocated from ER to cytoplasm, where they are degraded in a proteasome-dependent manner, a process dubbed ER-associated degradation (ERAD; [214]). Finally, to dispose of dysfunctional ER regions, an autophagy process can be engaged [215]. In *A. thaliana*, where UPR pathway has been extensively studied, all those strategies destined to relieve ER stress have been found conserved [200,216,217,218,219,220,221], with the notable exception of the UPR-contingent regulation of phospholipid synthesis that was barely addressed thus far [222].

Pioneering works have shown that chemically-induced ER stress triggers autophagic vesiculation in yeast [210,223] and mammalian models [187]. This process, named ER stress-Activated Autophagy (ERAA), was also reported as a backup system in animal cell cultures when ERAD was repressed by the use of proteasome inhibitors [224]. Likewise, in Arabidopsis, the occurrence of ERAA was probed using chemical, such as tunicamycin, an antibiotic that indirectly inhibits Asn-linked glycosylation of nascent polypeptides at the ER, and dithitreitol (DTT), a powerful reducer that interferes with disulphide bond formation. Again, these proteo-toxic treatments were demonstrated to promote an ER-phagy process that engulfs ER fragments and is completed by vacuolar digest [200,219]. Despite these similarities, molecular mechanisms underpinning autophagy activation by UPR differ between kingdoms. In mammalian cells undergoing ER stress, the transmembrane ER-resident kinase PERK is known to facilitate selective translation of the ACTIVATION TRANSCRIPTION FACTOR 4 (ATF4), responsible for a transcriptional reorientation that contributes to autophagy induction [208]. Another ER-localized actor of UPR, IRE1, can also provoke autophagy by means of protein interactions that switch on the JNK pathway [225]. No PERK orthologs could be identified in plants [226], and works conducted on Arabidopsis documented that ERAA is strictly controlled by one of the three Arabidopsis IRE1 proteins, i.e., IRE1B [219,227]. IRE1 are conserved multi-functional proteins composed of a N-terminal luminal sensing domain, a transmembrane followed by two successive cytoplasmic domains bearing kinase and ribonuclease activities, respectively. Under stressful conditions, IRE1 can self-dimerize and trans-phosphorylate, leading to ribonuclease activation [228]. Among Arabidopsis IRE1 ribonuclease targets are the bZIP60 TF-encoding mRNAs, which can be processed by unconventional splicing of their last introns. This event occasions a frameshift allowing to translate a soluble, active bZIP60 protein that can migrate to the nucleus, and participate in pro-adaptive UPR [217,221]. Regulated-IRE1-Dependent Decay of mRNA (RIDD) is another mode of action for those proteins, when they laterally cluster within ER membrane upon severe proteo-toxic stress. This results in degradation of ER-tethered mRNA [213,220,229], contributing to relieve unfolded protein pressure in lumen by limiting translation. In *A. thaliana*, the bZIP60 TF and the kinase activity of IRE1B were found dispensable for ERAA [219,227]. By contrast, recent investigations provide evidence for the involvement of IRE1B-mediated RIDD in this context [227]. This study supports the facts that (i) three RIDD targets could likely repress autophagy under restful conditions via a yet-to-deciphered mechanism, and (ii) this inhibition could be released by IRE1B through the degradation of the corresponding mRNA in response to tunicamycin. Negative regulators of AtIRE1B were also identified lately. The GOLGI ANTI-APOPTOTIC PROTEINS 1 and 3 (GAAP1 and 3), and the MEMBANE-ASSOCIATED PROGESTERONE RECEPTOR 3 (MAPR3) can all physically interact with each other, but also with IRE1B for modulating its RIDD activity in the recovery phase following ER stress [230,231]. Combined with previous data [227], these findings strongly suggest that the tryptic complex GAAP1/3 and MAPR3 could be involved in fine-tuning IRE1B-contingent RIDD, thereby accommodating ERAA levels during and after ER stress.

Beyond the mechanistic differences in UPR-initiated autophagy across kingdoms, the cyto-protective function of this process seems conserved [59,232,233]. In plants, like in mammals, sustained proteo-toxic ER stress seems to induce a selective autophagy that dispose of specific spatial ER domains. Recent works identified three Arabidopsis receptors for ER-phagy, namely the Sec62 translocon protein [234], C53 reticulophagy receptor [200], and Rtn2 reticulon isoform [235]. Consistently, *sec62* and *c53* mutants, like the *atg5* mutant, showed higher sensitivity to tunicamycin-induced ER stress when compared to wild-type plants [59,200,234]. Rtn2 binding to ATG8 was enhanced in response to DTT [235]. Bulk autophagy triggered by carbon starvation was independent on IRE1 proteins [219] whereas selective autophagy-induced by local inorganic phosphate depletion and heat shock required these UPR sensors to occur [59,236]. In line with these data and the idea that inducible autophagy can act as part of an endomembrane control quality system (Section 4.1), it is tempting to extend the hypothesis of Bernales and coworkers proposing the ER-phagy as a mechanism dedicated to counterbalance ER membrane expansion. One can imagine that ER-phagy, while recycling proteins and lipids, may also indirectly control lipid neo-synthesis through the specific elimination of discrete ER domains containing lipid biosynthesis complex [210].

Noteworthy enough, the mammalian UPR pathway itself is directly involved in many aspects of lipid metabolism. The three ER stress transducers, IRE1α, PERK, and the ACTIVATION TRANSCRIPTION FACTOR 6 (ATF6) can have both specific and overlapping roles in lipid homeostasis by regulating the Kennedy and mevalonate pathways, the lipolysis and lipogenesis, the TAG synthesis, the FA elongation, desaturation and oxidation, and the recycling of low-density lipoprotein receptor; though IRE1α is the one to display the broadest function in this context (see [237] for a recent review).

In plants, little is still known with regards to a potential direct link between lipids and UPR. Yet, a seminal study has reported that the maize *floury endosperm-2* mutant, expressing a mutated ER-resident protein that remains attached to the membrane, exhibited a constitutive ER stress associated with TAG and phospholipid over-accumulation in seeds [238], indicative of a causal role for this proteo-toxic stress in lipid changes. More recently, Arabidopsis phospholipid biosynthesis genes were proven misregulated in response to transient ER stress, but these variations in expression were not accompanied by any compelling glycerolipid phenotype [222]. The authors concluded on an active transcriptional regulation to maintain glycerolipid levels under stressful conditions. Yamaoka and collaborators also investigated IRE1 orthologous branch of UPR in the microalgae *Chlamydomonas reinhardtii* [239]. It was demonstrated that (i) CrbZIP1 was activated upon ER stress by the same mechanism as its Arabidopsis ortholog bZIP60, (ii) CrbZIP1 TF controlled the expression of a FA desaturase-encoding gene, and (iii) tunicamycin-challenged *crbzip1* knock-out mutants had an altered glycerolipid profile, specific of TAG and diacylglycerol-trimethylhomoserine (DGTS). Altogether, these results suggest that plant cells could employ UPR for adjusting lipid contents under ER stress. However, this question, which still awaits further investigations, would be complicated to address because of the intricate interdependency of UPR and autophagy.

Regulatory interactions between UPR and lipids can also be considered the other way around since membrane perturbations can represent an activation trigger for UPR in yeast and animal cells [206]. This facet of the literature is well-documented. It is known, for instance, that genetic manipulation of the *STEAROYL-CoA DESATURASE 1* (*SCD1*) can increase phospholipid saturation and launch UPR in mouse and HeLa cell cultures [240,241], and in the model worm *Caenorhabditis elegans* [242,243]. As elegantly demonstrated for the human UPR transducers PERK and IRE1α, the membrane-spanning domains of the two proteins were necessary for their respective self-assembly both in vivo and in vitro whereas their luminal unfolded protein-sensing domains were not required [241]. In line with this finding, the membrane saturation-induced UPR could be rescued by culture medium repletion with unsaturated FA [240,242]. Combined, these works thus indicate that UPR transducers can sense lipid-driven perturbations within ER membranes, independently from the organelle proteostasis status. Several lines of indirect evidence support the conservation of one such strategy in Arabidopsis. Firstly, mutants defective in the ER-resident *FATTY ACID DESATURASE 2*-encoding gene showed a marked decrease in polyunsaturated FA esterified on phospholipids, and this phenotype was correlated with a hypersensitivity to tunicamycin treatment [244]. This loss of ER stress tolerance was also reported for another phospholipid biosynthesis-deficient mutant [245]. Secondly, an EMS-based genetic screen identified a plastidial stearoyl-acyl carrier protein desaturase, responsible for the conversion of stearic acid (18:0) into oleic acid (18:1), as an upstream regulator of UPR [246]. Thirdly, glycerol application to seedlings, that was shown to augment steady-state levels of saturated lipids, was able to induce RIDD, but bZIP60 splicing, in *ire1* double mutant background engineered for expressing a luminal domain-deleted IRE1B protein [247]. Therefore, it is likely that lipo-toxic ER stress could actuate a protective RIDD response in Arabidopsis. However, it is still unclear whether this response could engage ER-phagy, as reported for proteo-toxic ER stress [219].

In conclusion, numerous biotic and abiotic stresses are believed to induce an ER stress in plants (see [248,249] for reviews), but the proteo- or lipo-toxic nature of those stresses has been poorly documented thus far. Nevertheless, based on the current literature, it is clear that ER-phagy, besides its recently proposed function in constitutive ER-QC via the rescue system of stalled-ribosomes [200], represents one cognate effector arm of the RIDD response. This protective arm is deployed in cases of artificially-induced severe ER stress, and its activation was also reported under heat shock [59] and inorganic phosphate deficiency [236]. It is also suspected to take place during senescence [191]. ER-phagy likely helps relieving ER stress by selectively engulfing organelle portions that are dysfunctional or became dispensable under such circumstances. Conceptually, one can thus envisage that this process could either contribute to lipid recycling over the stress period or aliment membrane remodeling during the recovery phase.

## 5. Conclusions

In plants, the nature, dynamics, and molecular functions of membrane lipids in autophagy remain poorly explored. Yet, recent research in other organisms has started to unravel their critical roles in AP formation by providing the building material for the formation and elongation of autophagic membranes, regulating their architecture and modulating the recruitment of the autophagy machinery. Distinct autophagy pathways are induced by different types of stress, targeting specific cargo, in selected plant organs or cell types. These lead to an adapted cellular and physiological response mediating plant acclimation and tolerance to environmental constraints. In that context, future research should focus on the understanding of how membrane lipids instruct plant autophagy, how they are mobilized to support acute induction and sustained rates of autophagy activity, and how these processes are integrated and differentially regulated upon varying environmental and developmental signals. We hope that this research will lead to novel concepts and tools that will help manage the challenge that global environmental changes impose on crops and biodiversity.

## Data Availability

Not Applicable.

## Supplementary Materials

The following are available online at https://www.mdpi.com/article/10.3390/cells10061272/s1, Table S1: List of studies in autophagy mutants highlighting the importance of autophagy in plant responses to stresses.
